# Effect of Extraction Methods on the Antioxidant Potential and Cytotoxicity of the Combined Ethanolic Extracts of *Daucus carota* L., *Beta vulgaris* L., *Phyllanthus emblica* L. and *Lycopersicon esculentum* against Gastric Adenocarcinoma Cells

**DOI:** 10.3390/molecules28186589

**Published:** 2023-09-13

**Authors:** Mahima Chauhan, Vandana Garg, Ghazala Zia, Rohit Dutt, Badrah S. Alghamdi, Ayat Zawawi, Ghulam Md. Ashraf, Aisha Farhana

**Affiliations:** 1 Department of Pharmaceutical Sciences, M.D University, Rohtak 124001, India; mahimachauhan708@gmail.com (M.C.); vandugarg@rediffmail.com (V.G.); ghazalazia94@gmail.com (G.Z.); 2 Rohit Dutt Principal, GMN College, Ambala 133001, India; rohitdatt23@rediffmail.com; 3 Department of Physiology, Faculty of Medicine, King Abdulaziz University, Jeddah 21589, Saudi Arabia; basalghamdi@kau.edu.sa; 4 Pre-Clinical Research Unit, King Fahd Medical Research Center, King Abdulaziz University, Jeddah 21589, Saudi Arabia; 5 Department of Medical Laboratory Sciences, Faculty of Applied Medical Sciences, King Abdulaziz University, Jeddah 21589, Saudi Arabia; atzawawi@kau.edu.sa; 6 Vaccines and Immunotherapy Unit, King Fahd Medical Research Center, King Abdulaziz University, Jeddah 21589, Saudi Arabia; 7 Department of Medical Laboratory Sciences, College of Health Sciences, and Research Institute for Medical and Health Sciences, University of Sharjah, Sharjah 27272, United Arab Emirates; 8 Department of Clinical Laboratory Sciences, College of Applied Medical Sciences, Jouf University, Aljouf 72388, Saudi Arabia

**Keywords:** hot-air oven drying method, ethanolic fruit extracts, bioactive compounds, flavonoids, carotenoids, phenolics, lyophilization method, phytoconstituents, antioxidant, anticancer

## Abstract

Frequent consumption of fruits and vegetables in the daily diet may alleviate the risk of developing chronic diseases. *Daucus carota* L. (carrot), *Beta vulgaris* L. (beetroot) *Phyllanthus emblica* L. (amla), and *Lycopersicon esculentum* M (tomatoes) are traditionally consumed functional foods that contain a high concentration of antioxidants, ascorbic acid, polyphenols, and numerous phytochemicals. This study assessed how three distinct preparation methods affect the phenolic, flavonoid, carotenoid, and ascorbic acid contents, antioxidant level, and cytotoxicity of the combined fruit extract. The fruit samples were taken in the ratio of carrot (6): beetroot (2): tomato (1.5): amla (0.5) and processed into a lyophilized slurry (LS) extract, lyophilized juice (LJ) extract, and hot-air oven-dried (HAO) extract samples. The sample extracts were assessed for their phytoconstituent concentrations and antioxidant and cytotoxic potential. The total phenolic content in LS, LJ, and HAO extracts was 171.20 ± 0.02, 120.73 ± 0.02, and 72.05 ± 0.01 mg gallic acid equivalent/100 g, respectively and the total flavonoid content was 23.635 ± 0.003, 20.754 ± 0.005, and 18.635 ± 0.005 mg quercetin equivalent/100 g, respectively. Similarly, total ascorbic acid content, carotenoids, and antioxidant potential were higher in the LS and LJ extracts than in HAO. Overall, the LS extract had a substantially higher concentration of phytochemicals and antioxidants, as well as higher cytotoxic potential, compared to the LJ and HAO extracts. The LS extract was tested in the MKN-45 human gastric cancer cell line to demonstrate its effective antioxidant potential and cytotoxicity. Hence, lyophilization (freezing) based techniques are more effective than heat-based techniques in preserving the phytoconstituents and their antioxidant and cytotoxic potential.

## 1. Introduction

The importance and awareness of proper food composition for a healthy life and disease prevention have grown in recent decades, leading to increased demand for functional foods. Beneficial nutrients in plant-based foods include dietary fibers, vitamins, minerals, and electrolytes. Essential phytochemicals like polyphenols, carotenoids, betalains, organosulfur compounds, alkaloids, etc., including numerous antioxidants, are also abundant in fruits and vegetables. The nutrient content of fruits and vegetables provide health benefits, while phytochemicals, especially polyphenols, have therapeutic potential [1]. Polyphenols are considered secondary metabolites that do not confer metabolic functions but are necessary for the nutritional and sensory qualities of plant foods [2,3,4]. The need of the hour is to use these phytonutrients as therapeutic agents against various diseases. Compared with synthetic chemotherapeutics, natural products such as anthocyanins and ginkgo biloba are proven to be less toxic to healthy cells, with fewer side effects in patients [5,6]. The literature is rife with efforts toward analyzing natural plant-based products as drugs against diseases, including cardiovascular diseases, cancers, inflammatory diseases, and autoimmune and infectious diseases [7,8,9,10,11]. Many phytonutrients have been in clinical trials, and some are being used in clinical settings [9,12,13,14].

Extraction of phytochemicals requires appropriate drying methods to preserve and retain these essential constituents from the fruits, leaves, or other parts of the plants. Numerous drying methods have been experimented with to preserve phytoconstituents. Some methods are sun drying, hot-air oven drying, freeze drying, vacuum drying, microwave drying, etc. Hot-air or conventional drying is the most popular method due to its low cost [15]. However, it has several disadvantages attributed to its long drying time and high temperatures, usually associated with undesirable effects, such as degradation of bioactive components, loss of antioxidant compounds and nutritional and sensory quality, higher shrinkage percentage, and the formation of undesirable secondary compounds [16,17]. Thus far, freeze-drying (lyophilization) is considered the best drying method that preserves dried foods’ sensory and nutritional qualities with lower shrinkage percentage, higher rehydration capacity, and easy application [18,19]. According to recent findings from evaluations of the effect of different drying techniques on major antioxidants in fruits and vegetables, freeze-drying outperforms other drying methods in terms of antioxidant preservation [20,21,22,23].

Due to the presence of phytochemicals, fruits, fruit juices, and vegetables demonstrate abundant synergistic effects on human health. In this study, we chose fruits of four plants: carrots, beetroot, tomatoes, and amla. Carrot was included due to its unique combination of three flavonoids: kaempferol, quercetin, and luteolin [24,25,26]. Beetroot contains phenolic compounds, betalains, carotenoids, micronutrients, and macronutrients [27]. The active compounds (betalains) in beetroot are a promising alternative for supplemental therapies for multiple diseases [28]. Numerous studies demonstrate that a high intake of lycopene-rich tomatoes and tomato-based products may protect against cardiovascular disease and reduce the risk of cancers of the prostate, breast, lung, and digestive tract [29]. Amla, which contains high concentrations of ascorbic acid, aids in immune defense, fights free radicals, and protects from various chronic diseases by neutralizing oxidative stress [30]. Carrots, beetroot, tomato, and amla have flavonoids, chlorogenic acid, and vitamin C, which positively impact human health through their protective effect against oxidative stress by neutralizing free radicals [31].

Many studies have reported the effect of different drying techniques on the antioxidant activity, physicochemical properties, and active concentrations of phenolic and other nutritional compounds of carrot, beetroot, tomato, and amla individually [32]. However, thus far, no research has been conducted on combined extracts of the above four fruits and the influence of different drying methods on the antioxidant capacity, total phenolic content, flavonoids, carotenoids, and ascorbic acid content, as well as their anticancer/cytotoxic activity [33,34].

## 2. Results and Discussion

### 2.1. Effect of Drying Techniques on Crude Fiber Content

**Figure 1:**
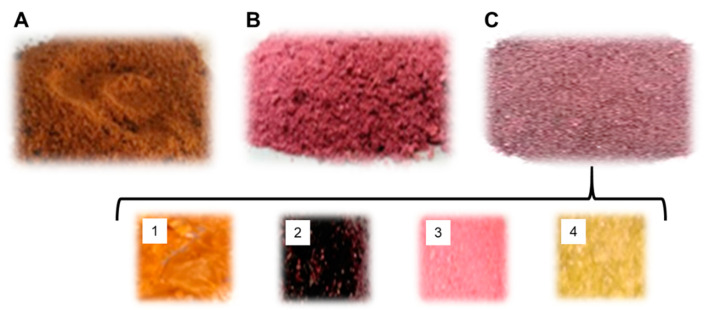
The dried extracts obtained from three methods used in the study. (**A**) The dried fruit sample obtained from the hot-air oven-dried (HAO) method. (**B**) The dried sample obtained from lyophilized slurry (LS). (**C**) The dried sample obtained from lyophilized fruit juices (LJ). (Inset) Examples of the dried juice extracts from each fruit (1—carrot; 2—beetroot; 3—tomato; 4—amla).

The samples were dried by the hot-air oven and lyophilization methods and mixed in the defined ratio as mentioned in the Materials and Methods section, i.e., carrot (6 mg): beetroot (2 mg): tomato (1.5 mg): amla (0.5 mg). The samples thus formed were; (A) hot-air oven-dried extract (HOA), (B) lyophilized slurry (LS) extract, and (C) lyophilized juice (LJ) extract, as shown in Figure 1.

The qualitative standard was determined using the HAO extract (Figure 1A) and lyophilized extracts (LS and LJ) (Figure 1B,C). The crude fiber content in LS, LJ, and the HAO extracts was 4.2%, 1.4%, and 2%, respectively, as shown in Table 1. The lowest value for crude fiber was obtained in the hot-air oven-dried preparation (HAO), and the highest crude fiber content was present in the LS extract. This indicates that the lyophilization methods have a less adverse effect and maintain the sample extracts’ fiber content. This was consistent with the findings of other, similar studies in which enzyme activities of the extracts were tested and found to be optimal with the lyophilization-based techniques [35,36]. High fiber content is beneficial in aiding the peristalsis movement of GIT, which helps bowel movement, lowers blood cholesterol, and reduces the risk of colon cancer [37].

### 2.2. Total Phenolic Content (TPC) and Flavonoid Content

Total phenolic and flavonoid content in the LS, LJ, and HAO extracts were determined (Table 2). The total phenolic content in the HAO extract was found to be low (72.05 ± 0.01 mg gallic acid/g) as compared to the LS extract (171.20 ± 0.02 mg gallic acid/g) and LJ extract (120.73 ± 0.02 mg gallic acid/g). Total flavonoid content in the LS extract (23.635 ± 0.003 mg quercetin/g) and LJ extract (20.754 ± 0.0005 mg quercetin/g) was found to be higher than in the HAO extract (18.635 ± 0.0005 mg quercetin/g). This indicates that the lyophilization (freezing) method is more resilient in preserving the TPC and flavonoid content than the hot-air drying method. It has been noticed in several studies that HAO leads to a loss of phytoconstituents, which potentially diminishes the bioactive value of functional foods [38]. Hence, an effective standardized drying method must be used to harness the full potential of fruit extract for health benefits or as therapeutics.

### 2.3. Ascorbic Acid Content and Carotenoids Content

The drying method employed to generate plant extracts influences the amount of ascorbic acid and carotenoids. Ascorbic acid is a hydrophilic, heat-sensitive vitamin easily destroyed and evaporated upon heating. As a result, the vitamin C content is usually lost during heat-based sample drying methods. Low temperature-based sample processing techniques are more promising to preserve and retain vitamin C and other nutrients during drying [39,40]. In our study, the ascorbic acid and carotenoid contents were more concentrated after lyophilization drying than with the hot-air oven drying method (Table 3). The concentration of ascorbic acid obtained in the samples dried using the hot-air method was found to be 2.68 mg. In comparison, LS and LJ extracts yielded 6.51 mg and 2.99 mg of ascorbic acid, respectively. Since fruit juices are prepared by extracting the liquid component from the slurry, low amounts of ascorbic acid in fruit juices, comparable to HAO, are probably due to a loss of ascorbic acid content upon filtration to remove the slurry. Secondly, ascorbic acid is more prone to oxidation in liquid solvents than in slurries. The carotenoids content in LS and LJ extracts was 30.25 mg/100 g and 23.25 mg/100 g, respectively, and 14.00 mg/100 g in the HAO extract (Table 3).

The nutritional profiles of plant extracts obtained after extraction limit or enhance their health benefits or their use as therapeutics. The research focus has shifted to identifying the best preparation methods that retain and enhance the bioactive contents, especially the antioxidant potential of fruit and vegetable extracts [41,42,43].

### 2.4. Antioxidant Activity

**Figure 2:**
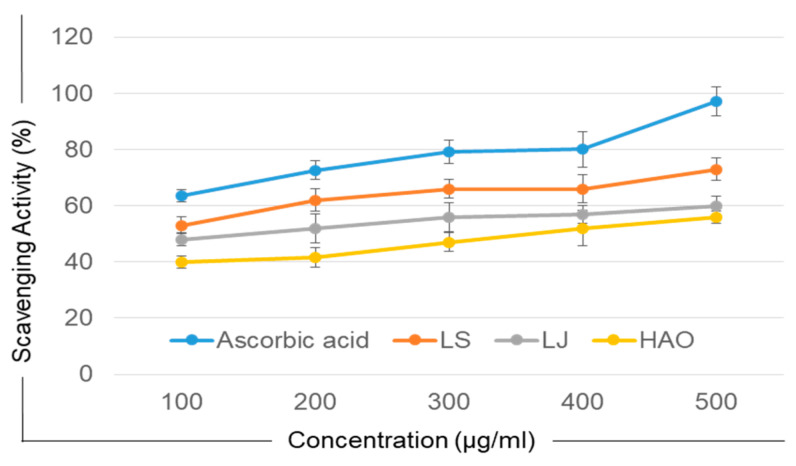
Free radical scavenging (antioxidant) activity using DPPH. The scavenging capacity of each dried sample extract (hot-air dried, lyophilized slurry, and lyophilized fruit juices) was assessed using DPPH. Ascorbic acid was used as a control. A high radical scavenging activity was observed in the lyophilized slurry compared to lyophilized juices or hot-air oven-dried samples.

The effect of different drying techniques on the antioxidant capacity of the HAO, LS, and LJ samples was evaluated using the DPPH scavenging method and reducing power assay. Table 4 shows the antioxidant activity of the three extracts. The percentage of inhibition was found to be highest in the LS at all concentrations compared to the LJ and HAO extracts. The results are presented in Figure 2.

The reducing power of all three extracts, along with ascorbic acid, is shown in Table 4. In the reducing power assay, the absorbance of the sample extracts reflected their reducing power or antioxidant capacity. The LS extract showed enhanced reducing power compared to the HAO and LJ extracts. An understanding developed from previous studies indicates that, in general, lyophilization methods provide effective preservation of chemicals and enhanced bioactivities, as compared to other methods that require dehydration-based high-temperature preservation and drying [39,44,45].

### 2.5. Anti-Proliferative Activity of Lyophilized Slurry (LS) Extract Assessed by MTT Assay

**Figure 3:**
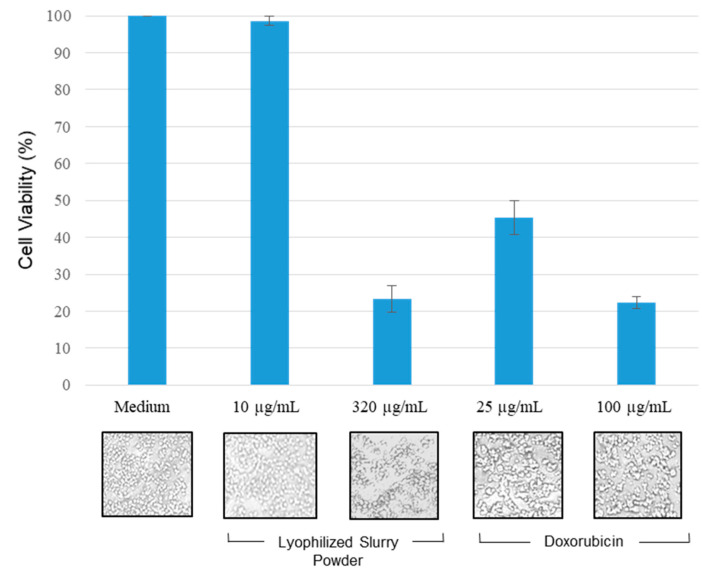
Inhibitory action (anti-proliferative potential) of lyophilized slurry (LS) extracts on MKN-45 cell line. MKN-45 cells were incubated for 24 h with LS at 10 μg/mL and 320 μg/mL concentrations and doxorubicin at 25 μg/mL and 100μg/mL concentrations. Percentage cell viability was tested using an MTT solution. The histograms depict the percentage viability of the MKN-45 cells treated with ethanol, LS extract, and doxorubicin after 24 h exposure to the above-mentioned concentrations. The data are presented as mean ± SD for three replicates.

In vitro results indicate that the LS extract had a higher concentration of phytoconstituents and higher antioxidant activity than the HAO and LS extracts (Table 2 and Table 4). Hence, we chose the LS extract to test the anti-proliferative (anticancer) activity using a gastric adenocarcinoma cancer cell line (MKN-45). Doxorubicin was taken as a control to compare the efficacy of the LS extract on MKN-45 cells. Two concentrations of doxorubicin (25 µg/mL and 100 µg/mL) and LS (10 µg/mL and 320 µg/mL) were used. As evident from the cytotoxicity assay, the LS extract showed considerable cytotoxicity with a 320 µg/mL concentration (23.33 ± 3.5% viability), which was similar to that of doxorubicin (22.11 ± 1.5% viability) at a much lower concentration of 100 µg/mL (Figure 3).

Notably, previous studies demonstrated that single fruit extracts prepared by ethanol extraction had higher IC_50_ values than the combined fruit extract (LS) in our study. Ethanolic amla extract demonstrated an IC_50_ of up to 650 µg/mL tested against various cancer cell lines [46]. Similarly, Convolvulus pluricaulis leaf extract showed an IC_50_ value of approximately 1000 µg/mL in HepG2 and L-929 cell lines [47]. Nonetheless, some reports of the synergetic effect and the increased potency of combined extracts are available [48,49]. Due to the enhanced overall health benefit, recently many researchers have shifted their focus from an interest in extracted and purified single components to using combined fruit extracts for their antimicrobial, antioxidant, and anti-cancer properties [50].

Studies have also reported that mixing and blending bioactive fruits stabilizes the total phytoconstituents [51]. Recently, scientific research on combined extracts has shed light into their enhanced bioactivity. Hence, this study is among the few upcoming studies that can help to develop a better understanding of the combined fruit extracts and extraction methods aimed at enhancing the overall beneficial effects of fruits.

## 3. Materials and Methods

### 3.1. Sample Collection

Carrots, beetroot, tomatoes, and amla were collected from the local food market and authenticated by the quality assurance authority of Maharishi Dayanand University, Rohtak, Haryana, India.

### 3.2. Reagents

All chemicals, reagents, and solvents of analytical grade (AR) were purchased from Loba Chemicals Pvt. Ltd. Mumbai, India, Himedia (HiMedia Laboratories, Mumbai, India), CDH Fine Chemicals (Central Drug House (P) Ltd., Gujarat, India), and Merck (Branchburg, NJ, USA).

### 3.3. Drying Processes

**Figure 4:**
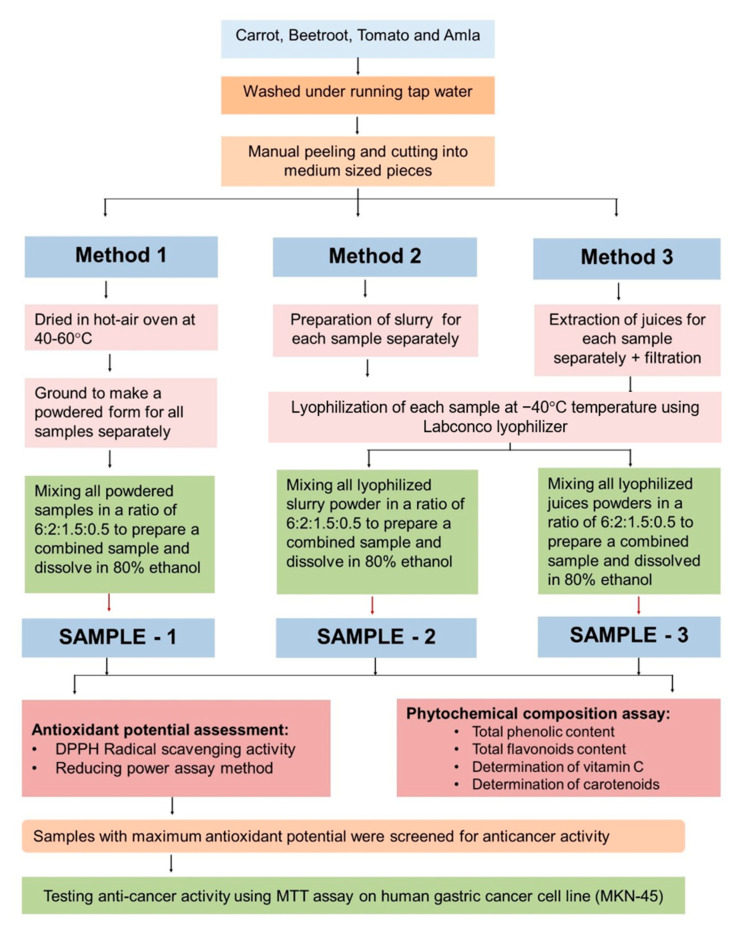
Flowchart of the methodology used in the study. The step-by-step methodology starting from sampling to sample drying, extract preparation, and sample testing, is shown in the flowchart.

Fresh ripe carrots, beetroot, amla, and tomatoes were purchased from the local food market. Each sample was washed, sliced into smaller pieces, and juiced independently before being dried through different methods. We prepared three samples, *viz.* lyophilized slurry extract, lyophilized juice extract, and hot-air oven-dried extract, using freeze-drying or heat-drying techniques. Two methods, slurry or juice, that were subsequently freeze-dried, were used. The sample collection, preparation, and testing methodology are explained in the flowchart (Figure 4).

### 3.4. Preparation of Samples

After adequately washing the beetroots, carrots, tomatoes, and amla under running water, the peels were removed from the fruits except for the amla and tomatoes. Amla was stripped of its seeds and chopped into medium-sized pieces.

#### 3.4.1. Method 1 (Hot-Air Oven-Dried Powder Sample)

Each fruit sample was dried in a hot-air oven at 40–60 °C temperature, ground individually, and then mixed in a ratio of carrot (6 mg): beetroot (2 mg): tomato (1.5 mg): amla (0.5 mg). The samples were stored in an airtight container until further use (Figure 1). The temperature range for hot-air drying was set to ensure that fruits with high moisture content (tomatoes and amla) were dried completely, similarly to that of beetroots and carrots. The 40–60 °C temperature range has been optimized for drying all four fruits to a final moisture content of 0% [52]. Secondly, the ratio (carrot (6 mg): beetroot (2 mg): tomato (1.5 mg): amla (0.5 mg)) was also optimized as the highest in antioxidant potential among the three ratio combinations tested.

#### 3.4.2. Method 2 (Lyophilized Slurry Powder Sample)

Each fruit sample was separately put into a grinder to produce a slurry. The slurries were lyophilized at −40 °C temperature using a Labconco lyophilizer. Lyophilized extracts were again mixed in the same ratio as mentioned in Method 1, i.e., carrot (6 mg): beetroot (2 mg): tomato (1.5 mg): amla (0.5 mg), and stored in an airtight container until further experimentation.

#### 3.4.3. Method 3 (Lyophilized Juice Powder Sample)

Each fruit sample was separately put into a grinder to produce a slurry and subsequently filtered to obtain the juice. The fruit juices were lyophilized separately at −40 °C temperature using a Labconco lyophilizer. Lyophilized juice powders were mixed in the same ratio as mentioned in Method 1, i.e., carrot (6 mg): beetroot (2 mg): tomato (1.5 mg): amla (0.5 mg) and stored appropriately until further use.

### 3.5. Extraction

The three dried (powder) extracts—HOA extract, LS extract, and LJ extract—were prepared through an ethanol-based extraction procedure. A quantity of 100 mg of dried (powder) sample (prepared using method 1, method 2 or method 3) was soaked in 500 mL of 80% ethanol/water in tightly covered bottles and left for 24 h with occasional mixing. The ethanol extracts were centrifuged at 2500 rpm for 10 min at 25 °C and were subsequently filtered using Whatman filter paper No. 1 (Whatman International, Maidstone, UK). The samples were further evaporated to dryness at 50 °C using a rotary evaporator until concentrated extracts were obtained. Each extract was weighed and diluted with specific solvents (according to the solvent required for each experiment) to prepare different concentrations to be used for further experiments. The extracts were either used for in vitro studies immediately or stored under sterile conditions at −20 °C until further use (with slight modification from Lim, 2019).

### 3.6. Determination of Crude Fiber Content

Crude fiber content was determined for all three extracts using the standard procedure according to the Indian Pharmacopoeia 2018 (https://www.webofpharma.com/2022/04/indian-pharmacopoeia-2018-ip-2018-pdf.html, accessed on 7 October 2021) (Indian Pharmacopoeia, 2018) [53].

### 3.7. Determination of Total Phenolic Content

The phenolic contents (free and bound phenols) were analyzed by spectrophotometer using the Folin–Ciocalteu (FC) reagent method. All three dried extracts were dissolved in methanol, mixed with 125 µL of FC reagent, and left to stand for 6 min. After that, we added 1.25 mL of 7% Na_2_CO_3_ solution, and the final volume was made up to 3 mL using distilled water [54]. The samples were left at room temperature for 90 min, and absorbance was noted at 760 nm using a UV/Vis spectrophotometer. The linearity reading of the standard curve was measured with gallic acid/100 g used as standard [55].

### 3.8. Determination of Total Flavonoid Content

A volume of 0.3 mL of 5% NaNO_2_ solution was mixed with 1 mL ethanolic extract for all three samples separately. After 5 min of incubation, we added 0.3 mL of 10% AlCl_3_ and incubated the mixture of samples at room temperature for 6 min. Then, we added 10 mL of 1 M NaOH solution. Finally, the reaction mixtures were left in a dark place for 15 min. We took 0.3 mL volumes from these solutions and measured the absorbance of the samples at 510 nm using a UV-Vis spectrophotometer. Quercetin was used as a control, and the results expressed in mg of quercetin equivalent (QE) per g of dried extracts [56].

### 3.9. Determination of Total Ascorbic Acid

A 0.005 mol L^−1^ iodine solution and 0.5% starch indicator solution were prepared separately to determine ascorbic acid in the prepared samples. A 20 mL aliquot of the sample solutions was taken into a 250 mL conical flask. Further, 150 mL of distilled water and 1 mL of starch (indicator) solution were added. The three samples were titrated using 0.005 mol L^−1^ iodine solution [57]. The endpoint of the titration was identified with the appearance of a dark blue-black color due to the formation of the starch–iodine complex [58].

The ascorbic acid concentration in the three extracts was calculated as follows:

Ascorbic acid (mg) = M _iodine solution_ × mL _iodine solution_ × 176.12 g/mol

### 3.10. Extraction and Determination of Carotenoids

Two grams of the LS, LJ, and HAO extracts was dissolved in acetone and hexane (1:1) solution. Five milliliters of acetone was added to each 2 mL extract after specific time intervals, and this process was repeated two to three times. Each acetone-washed extract was filtered and collected separately into a beaker. The collected extracts were applied to a separating funnel, and a 10% NaCl solution in distilled water was passed over the extract through the separating funnel. The resulting solution was thoroughly stirred and kept aside for the separation of layers. Once the layers separated, the upper layer containing carotenoids was collected and treated to eliminate the water and NaCl (anhydrous) solution [59]. The absorbance of carotenoids in the obtained sample was recorded at 630 nm using a visible spectrophotometer, and the total amount of carotenoids in 100 g of each dried sample was calculated. Freshly made β-carotene standard solution of 2 mg/mL prepared in acetone was used as a standard.

### 3.11. 2,2-Diphenyl-1-Picrylhydrazyl Radical Scavenging Activity

2,2-Diphenyl-1-Picrylhydrazyl (DPPH) was used to assess free radical scavenging activities of the extracts. In 0.1 mL of each ethanolic extract (LJ, LS, and HAO extracts), diluted in 0.8 mL DMSO, 0.1 mL of DPPH in methanol was added. After incubating the samples for 30 min, the absorbance was measured at 517 nm. Following this method, a lowering of the absorbance reading shows higher free radical scavenging activity. The % scavenging activity of DPPH was calculated by the following equation:

% Inhibition = (A_control_ − A_sample_/A_control_) × 100

where A_sample_ is absorbance of sample solution, A_control_ is absorbance of control. DMSO was used as blank, ethanol was used as a control, and quercetin solutions were used as standards for the same run. The method is similar to previously established protocols [60,61].

### 3.12. Reducing Power Assay

One milliliter of each extract (LJ, LS and HAO) was combined with 2.5 mL of phosphate buffer (pH 6.6) and 2.5 mL of 1% potassium ferricyanide solution, followed by thorough mixing, and incubated for 20 min at 50 °C. After incubation, 2.5 mL trichloroacetic acid (TCA) was added, and the sample was centrifuged for 10 min at 3000 RPM. The supernatant was transferred into a different tube, and 2.5 mL of the supernatant was mixed with 2.5 mL of distilled water and 0.5 mL of 0.1% Iron (II) chloride (FeCl_2_). The absorbance of samples was observed at 700 nm against the blank (phosphate buffer) using a UV-Vis spectrophotometer. Ascorbic acid was taken as a reference standard, prepared similarly without the addition of a sample. An increase in the absorbance reading of the reaction mixture depicted an increase in the reducing power (Figure 2)

% Reducing Power = (1 − A_sample_/A_control_) × 100

A_sample_ is the absorbance of the sample solution, and A_control_ is the absorbance of control [18]. 

### 3.13. Evaluation of the Cytotoxic Activity of the LS Extract in Gastric Adenocarcinoma (MKN-45) Cell Lines by MTT Assay

**Figure 5:**
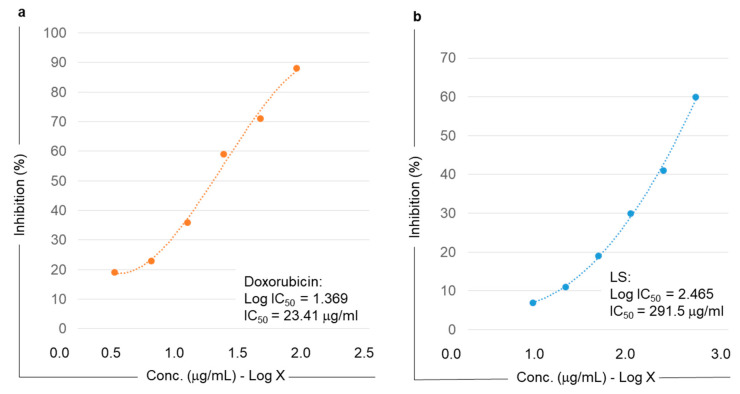
Calculation of IC_50_ values of the lyophilized slurry (LS) extracts. The sample extracts were tested for their cell proliferation inhibitory effect on human gastric cancer cell line MKN-45 using the MTT assay. (**a**) Doxorubicin was used as a standard and was assayed using the concentrations ranging from (0.5–2.5 μg/mL − log X), while (**b**) lyophilized slurry (LS) extract was tested in the range from 1 to 3 μg/mL (log X). The IC_50_ value was calculated using the standard formula.

The gastric adenocarcinoma cell line (MKN-45) was cultured under optimum conditions in DMEM media with 10% fetal bovine serum (FBS) and 5% CO_2_ until a bilayer cell density was achieved. The bilayer was trypsinized using culture media without FBS, and a 5.0 × 10^5^ cells/mL cell count was taken. One hundred microliters of diluted cell suspension (50,000 cells/well) was added to each well of the 96-well microtiter plate. After 24 h, a partial bilayer was reached. The supernatant was removed, and the bilayer was washed with culture media. The cells were then treated with 100 µL of different concentrations of the extract, diluted in DMEM medium. The plates were incubated for 24 h at 37 °C in a 5% CO_2_ atmosphere. After incubation, the sample solutions were removed from the wells, and 100 µL of MTT (5 mg/10 mL MTT in PBS) was added to each well. The plates were further incubated for 4 h. The supernatant was removed, and 100 µL of DMSO (dimethyl sulfoxide) was added to each plate well, which was gently agitated to dissolve the formazan formed by MTT. The absorbance was measured at a wavelength of 590 nm using a microplate reader [62]. The percentage growth inhibition was computed, and the dose–response curves were generated for each concentration of the extract to determine the concentration of the test sample required to inhibit cell growth by 50%. The concentration at which 50% growth inhibition was observed was considered the 50% inhibitory concentration (IC_50_) (Figure 5).

Calculating the inhibition percentage

% Inhibition = [(OD of Control − OD of Sample)/OD of Control] × 100

The IC_50_ concentrations were used to test the inhibitory effect of the extract on MKN-45 cell proliferation (Figure 3).

## 4. Conclusions

The total phenolic, flavonoid, carotenoids, and ascorbic acid concentrations were much higher in the LS extract than in the LJ and HAO extracts. In the LJ extract, moderate concentrations of these antioxidants were present. The HAO extracts had very low ascorbic acid concentrations and total phenolic, flavonoid, and carotenoids contents. The LS extracts also demonstrated higher antioxidant and anti-proliferative activity, possibly attributable to higher total phenolic, flavonoid, carotenoids, and ascorbic acid contents retained during drying.

Finally, based on the findings of our study, we confirm that drying methods substantially impact the stability, activity, preservation, and concentration of antioxidant and therapeutic phytochemicals present in plant products. Hence, drying techniques should be carefully and efficiently used to maintain and protect the therapeutic capacity of dried plant extracts to obtain significant health and treatment benefits.

## Figures and Tables

**Table 1:** Effect of three drying techniques on the crude fiber content.

Dried Samples	Weight (g)	Crude Fiber (%)
Hot-air oven-dried sample (HAO)	2.0	2.0
Lyophilized slurry (LS)	2.0	4.2
Lyophilized juices (LJ)	2.0	1.4

**Table 2:** Total phenolic content and flavonoid content in hot-air oven-dried extract, lyophilized slurry extract and lyophilized juice extract.

Dried Samples	Total Phenolic Content (mg Gallic Acid/100 g)	Total Flavonoid Content (mg Quercetin/100 g)
Hot-air oven-dried sample (HAO)	72.05 ± 0.01	18.635 ± 0.005
Lyophilized slurry (LS)	171.20 ± 0.02	23.635 ± 0.003
Lyophilized juices (LJ)	120.73 ± 0.02	20.754 ± 0.005

**Table 3:** Determination of ascorbic acid content and carotenoids in the three extracts.

Different Dried Samples	Ascorbic Acid Content (mg)	Carotenoids Content (mg/100 g Sample)
Hot-air oven dried sample (HAO)	2.68	14.00
Lyophilized slurry (LS)	6.51	30.25
Lyophilized juices (LJ)	2.99	23.25

**Table 4:** Determination of antioxidant activity of the three sample extracts.

Drying Techniques	Reducing Power (Total Antioxidant Capacity) (Absorbance)
Hot-air oven-dried sample (HAO)	0.235
Lyophilized slurry (LS)	1.827
Lyophilized juices (LJ)	1.521

## Data Availability

All data and materials used in this study are available from the corresponding author and will be provided upon reasonable request.
